# Cloning Coconut via Somatic Embryogenesis: A Review of the Current Status and Future Prospects

**DOI:** 10.3390/plants10102050

**Published:** 2021-09-29

**Authors:** Sundaravelpandian Kalaipandian, Zhihua Mu, Eveline Yee Yan Kong, Julianne Biddle, Robyn Cave, Amirhossein Bazrafshan, Kusinara Wijayabandara, Fernanda Caro Beveridge, Quang Nguyen, Steve W. Adkins

**Affiliations:** 1 School of Agriculture and Food Sciences, The University of Queensland, Gatton, QLD 4343, Australia; z.mu@uq.edu.au (Z.M.); eveline.kong@uq.net.au (E.Y.Y.K.); julianne.biddle@aciar.gov.au (J.B.); r.cave@uq.edu.au (R.C.); a.bazrafshan@uq.edu.au (A.B.); k.wijayabandara@uq.edu.au (K.W.); fernanda.carobeveridge@uq.net.au (F.C.B.); s.adkins@uq.edu.au (S.W.A.); 2 Australian Centre for International Agricultural Research, Canberra, ACT 2617, Australia; 3 Applied Biotechnology for Crop Development Research Unit, The International University, Ho Chi Minh City 700000, Vietnam; quang.nguyen212@gmail.com

**Keywords:** coconut, somatic embryogenesis, cloning, explants, plant growth regulators, medium, acclimatization, genetics

## Abstract

Coconut [*Cocos* *nucifera* L.] is often called “the tree of life” because of its many uses in the food, beverage, medicinal, and cosmetic industries. Currently, more than 50% of the palms grown throughout the world are senile and need to be replanted immediately to ensure production levels meet the present and increasing demand for coconut products. Mass replanting will not be possible using traditional propagation methods from seed. Recent studies have indicated that in vitro cloning via somatic embryogenesis is the most promising alternative for the large-scale production of new coconut palms. This paper provides a review on the status and prospects for the application of somatic embryogenesis to mass clonal propagation of coconut.

## 1. Introduction

Coconut [*Cocos nucifera* L.] is grown in most tropical and subtropical regions of the world. It offers food, shelter, and income to people via the hundreds of products it provides [1,2]. Demand for coconut products has increased up to five-fold in the last 10 years; however, production has not kept up with this growing demand [3]. Very little or no replanting of coconut palms has been undertaken in most production countries and there are now reports of regions carrying more than 70% senile palms [4]. Apart from senility, coconut production currently faces other constraints, including biotic and abiotic stresses, and a lack of quality planting materials from seednuts. Coconut is susceptible to pest and diseases such as the coconut rhinocerous beetle (*Oryctes rhinoceros* L.), the red palm weevil (*Rhynchophorus ferrugineus* Olivier), lethal yellowing diseases including Bogia coconut syndrome, and viroid diseases such as cadang-cadang, and climatic conditions such as drought intensified by recent climate change events [5]. Replanting efforts have been limited due to the lack of good quality and true-to-type planting materials. As Tall coconut types cross-pollinate, the establishment of new palms from seednuts may show a high degree of variation in fruit yield and other traits, which can be observed only at palm maturity [6]. In such Tall types, controlled pollination is required to produce true-to-type palms, and this is difficult to achieve on a large scale due to the height these palms can attain at maturity. In addition, when disease-resistant plants are identified, they are usually few in number and therefore unable to produce sufficient seednuts [7] to meet the high demand for disease-resistant seedlings. Similarly, some elite varieties cannot be grown from seed, such as the makapuno and aromatics [8,9,10]. Due to these constraints, large quantities of high-quality planting materials to meet the growing demand cannot be supplied by traditional means, and no vegetative propagation methods are available for plantlet production [9]. In vitro culture has the potential to rapidly multiply a chosen genotype to produce numerous clonal plantlets. Ideal application could result in the mass production of early-bearing, disease-free, and resistant palms that have high productivity or other valuable characteristics [5,11]. Somatic embryogenesis (SE) has been identified by many as the most feasible technique for large-scale production of high-quality coconut plantlets [12,13,14].

Coconut SE involves, firstly, the induction of embryogenic callus, the formation and development of the somatic embryo, its maturation, then germination, and finally the recovery of the plantlet formed [15]. During the process of SE, the dedifferentiated somatic cells regain their epigenetic and biochemical competence to form somatic embryos that progress through a series of developmental stages such as zygotic embryogenesis [16]. Somatic embryogenesis in the palm family was first described for oil palm (*Elaesis guineensis* Jacq.) by Rabechault et al. in 1970 [17], and later in coconut by Eeuwens and Blake, in 1977 [18]. To date, these techniques have been considerably improved to be able to produce coconut plantlets in vitro on a large scale [7,19].

At every stage in the SE approach, from somatic cells to whole plantlet formation, numerous factors have been found to play a crucial role in success, including the genotype of the donor plant, the explant type cultured, the media and plant growth regulators (PGR) used, and the acclimatization procedures applied to the germinated plantlets. Several coconut tissues have been used for explants *viz*., shoot tips, plumules, rachillae sections, young leaves, unfertilized ovaries, and immature embryos, with the response varying depending on the explant type [13]. Plant growth regulators used in the media are known to be another major influencer of the rate of success in the production of plantlets via SE. For example, a low concentration of 2,4-dichlorophenoxyacetic acid (2,4-D) is known to induce the best quantity of embryogenic cells in several Sri Lankan Tall varieties [20], whereas a high concentration of 2,4-D was required for Malayan Yellow Dwarf to achieve the similar results [21]. These heterogenous responses are an example of the challenges faced in coconut clonal propagation, which can be overcome by the selection of the most responsive explants, the best concentration of PGRs, and the most appropriate media components. In addition, the identification of genes that are involved in the process of SE is helping to facilitate the SE process in coconut. The present review summarizes the status of SE in coconut and identifies possible future developments to help meet the growing demand for plantlets to replant traditional and new coconut lands with elite varieties.

## 2. Importance of Suitable Explants

Cell totipotency is an important characteristic of plant cells, but not all cells are totipotent. The somatic cells that demonstrate a sensitivity to embryogenesis and are capable of undergoing SE are described as being totipotent. The capability of tissue to produce somatic embryos is mainly dependent on a restricted fraction of their cell population, rather than a distinct area within this cell population [22]. Hence, an explant with a source of totipotent cells is the key prerequisite for SE formation by external inducement. The SE response can also be influenced by the age of the explant. Various types of explants have been used for undergoing SE in other species, and include seedlings, leaves, petioles, shoot meristems, roots, seeds, cotyledons, zygotic embryos, and immature zygotic embryos [23].

Somatic tissues from the coconut, such as young leaves, stem sections from young seedlings, and tissues from immature inflorescences have all been studied as explants to generate the embryogenic callus of coconut. The culture of living tissues of coconut was first undertaken by Eeuwens and Blake, 1977 [18], who used seedling stem and immature inflorescence sections as explants. The first SE attempts on coconut explants showed a calloid-like structure to be formed, which is a more organized structure of the callus [24]. Under suitable conditions, plantlets can be produced from the somatic embryos that formed from this embryogenic callus [25]. Although this early approach was inefficient, the embryogenic callus induction rate was increased in later years [26]. In many species it is known that the selection of an explant from a healthy and vigorously growing plant is particularly important to obtain good tissue culture outcomes [27]. In coconut, the type and stage of explant development can significantly affect the responsiveness of the explant to tissue culture [28]. Somatic tissues, such as rachilla segments from immature explants, were commonly used in the early studies of coconut tissue culture because true-to-type clones could be produced from such tissues [7]. However, it is challenging to use these types of coconut tissues. For example, the correct age of the inflorescence tissue to be used as an explant can be difficult to determine [29], and harvesting this material is destructive if not fatal to the palm. Furthermore, the callus induction rate from inflorescence tissues is often lower than 30%, with poor repeatability [30,31].

Zygotic tissues such as embryos or plumules have also been used as explants in coconut SE work [13]. Adkins et al., 1999 [32] found that immature zygotic embryos were superior explant tissues for SE, with a callus induction rate of 50%, compared to just 3% from mature embryos. Similarly, Karunaratne and Periyapperuma 1989 [33] reported that embryos over 8 months of age were already undergoing germination and therefore they were not suitable for callus formation. Whole embryos, in addition to sections of embryos, have been used as explants [32,34]. For embryo sections, the middle portion was found to be the best for callus formation, ranging from 58% to 83% [32,35]. This may be because the central tissue contains much of the embryo axis. The callus induction rate was shown to be dramatically decreased when the surrounding, cotyledonary tissue was used [7,34].

More recently, plumular tissues from germinating zygotic embryos [11,36,37,38] and rachilla sections from immature inflorescences [39,40] have been successfully used for coconut SE. Of these, the plumular tissue was found to be the most suitable for SE [30,41,42,43]. Perez-Nunez et al., 2006 [11] hypothesized that 98,000 somatic embryos could be produced from a single zygotic plumule. The technique would need to use the repeated subculture of plumular callus, which could rapidly increase the total cell biomass and promote the formation of secondary somatic embryos [44]. Such an approach, however, may not produce true-to-type plantlets unless the self-pollinating Dwarf varieties of coconut are cultured, and the process may suffer from somaclonal variation if the number of callus subcultures is numerous.

Leaves from 5 year old coconut palms have also been used as explants, but the callus induction rate was very low, at less than 20%, and somatic embryos took up to 6 months to develop [45,46]. Shoot meristem explants isolated from zygotic embryos achieved a 79% callus induction rate [47]. However, the shoot meristem explant excised from the in vitro germinated embryos showed a lower percentage of SE callus formed, and this may be due to meristematic multiplication inhibition by the presence of cotyledonary tissues [47]. Although anthers have also been used as an explant source [14,48,49,50,51], the frequency of plantlet production was low, at only 0 to 7% from calli/embryos that were subcultured in SE induction and maturation medium [52]. Unfertilized ovaries have been used as another novel explant type for coconut SE, particularly as such somatic tissues would be capable of producing true-to-type plantlets from all coconut varieties. Using such explants, a callus induction rate of 41% [42] has been achieved with an efficiency of plantlet production of about 30%, resulting in 83 plantlets from 32 ovaries [52], which is still below what would be needed for a commercial application [53,54]. Thus, the results suggest that plumular tissues are the preferred explant type for large-scale production of Dwarf coconut types. However, further work is required on somatic tissues, such as ovaries and young inflorescence tissues, to enable the production of true-to-type clones from Tall coconut types. The studies on coconut explants and varieties are summarized in Table 1.

## 3. Effect of Media Composition

The growth and biomass accumulation of tissue culture plants is driven by the assimilation of carbohydrates, nitrogen, phosphate, magnesium, calcium, and other ions. The Y3 medium was specifically developed for coconut tissue culture [18] and is now widely used for most coconut work [64,65]. Within this medium, a high concentration of sucrose (usually > 4%) is used as the main carbohydrate source [64]. However, this high sugar content can result in an increased risk of microbial contamination, allowing the contaminants to compete with the explant for nutrients in the medium [66].

Activated charcoal (AC) is also commonly used in coconut tissue culture [67]. This substance has a matrix structure with very fine pores that gives it a large surface-to-volume ratio [68]. The main purpose of AC is to absorb and therefore remove inhibitory substances (e.g., phenolic compounds) that are produced by the explant tissue and that accumulate in the media. Variation in particle size of AC, which occurs between batches, can affect the frequency of SE callus formation [69]. For example, AC with a small particle size produced higher frequencies of embryogenic callus (70%) than large fractions or whole AC in coconut [69].

Most often, a solid medium is used for coconut culture work; however, a liquid medium has benefits, not only in the saving of cost and time, but also in giving the callus greater contact with the nutrient supply. The use of gelling agents and the precise positioning of explants on the medium surface are also not necessary when a liquid medium is used [70]. However, the application of a temporary immersion system, where the explants are immersed in a liquid medium for a specific time and duration per day, may improve growth of germinating somatic embryos, providing the benefits of both solid and liquid medium systems [65], and dramatically increasing the SE-generated plantlet rate for coconut. A liquid medium was used to produce viable cell suspension cultures in coconut; however, they were unable to progress to the next stage because it is challenging to produce friable embryogenic calli for SE initiation [47,71]. Table 1 summarizes the in vitro studies undertaken on media and media composition for the induction of callus in coconut.

## 4. Role of Plant Growth Regulators

Plant growth regulators are necessary to regulate callus formation, multiplication, somatic embryo formation, and their and germination responses. Although exogenously applied PGRs play a key role in these processes, the endogenous concentrations are also important for successful SE [22,23]. Thus, the interaction between exogenous and endogenous PGR concentrations during SE are important to its outcomes. In addition, the explant tissues sensitivity towards each specific group of PGRs determines SE outcomes [72]. For a wide range of plant species, over 80% of the protocols developed use auxins alone or in combination with a cytokinin to induce SE [23]. For coconut SE, embryogenic callus induction and proliferation can be promoted by a high concentration of auxin (2,4-D at 24 to 600 µM in the presence of AC), whereas a subsequent reduction in 2,4-D concentration has been shown to aid the maturation and germination of the somatic embryos formed [13]. Auxin (2,4-D) has also been used for the initiation of embryogenic cell suspension cultures in coconut propagated through the typical SE pathway [71], but only when provided at a very low concentration (4.5 µM) [47]. The concentration of 2,4-D required for callus induction varies between coconut varieties and for different explant types. Embryogenic callus induction from zygotic embryos required a lower concentration of 2,4-D (24 µM), whereas a high 2,4-D concentration (600 µM) was necessary for embryogenic callus induction from plumule and young inflorescence tissues [13]. A combination of a cytokinin, such as kinetin and 2iP (6-(γ,γ-Dimethylallylamino) purine; both at 10 µM), and 2,4-D (100 µM), have been found to increase the production of callus and somatic embryos from microspores in coconut [52]. Many studies have demonstrated that maturation of coconut somatic embryos will only occur after the reduction or withdrawal of 2,4-D from the medium [11,13].

Cytokinins, such as 6-benzylaminopurine (BAP), thidiazuron (TDZ), kinetin, or 2iP, are common additions to the medium for the maturation of coconut somatic embryos [13]. For example, Pérez-Núñez, et al., 2006 [11] used media supplemented with 6 µM 2,4-D and 300 µM BAP for the induction of primary somatic embryos on callus derived from plumule tissues. A combination of TDZ and 2,4-D has been shown to enhance coconut callus induction and to decrease tissue browning [28].

Gibberellic acid (GA_3_) interacts with many plant processes, including the regulation of proteins that result in changes in gene expression, cellular behavior, and metabolism [73]. According to Montero-Córtes et al., 2010 [74], GA_3_ (0.5 µM) improved the formation and germination of coconut somatic embryos, with the maturation rate increasing 1.5-fold, and the number of somatic embryos formed per callus clump increasing 2.0-fold. Gibberellic acid has also been included in a recent protocol developed for the germination of coconut somatic embryos derived from plumular [38] and inflorescence tissues [39] at concentrations of 2.8 and 4.6 µM, respectively.

Brassinosteroids promote cell division and differentiation of tissues of many species. They have been found to increase the production of primary callus, embryogenic callus, and somatic embryos from the plumular tissue of coconut [75]. Abscisic acid (ABA; 2.5 to 5.0 µM) has been shown to promote somatic embryo development in coconut [20,34], when applied to nodular calli derived from immature zygotic embryos [20,33].

The effect of PGRs on the maturation of somatic embryos can be influenced by other factors such as medium pH and osmotic stress [76]. For example, coconut somatic embryos, exposed to 30 g L^−1^ polyethylene glycol and 45 µM ABA, increased the rate of formation, maturation, and regeneration [34]. This treatment also improved the morphological appearance of the somatic embryos and promoted more synchronous growth. The combination of ABA (10 µM) with amino acids such as glutamine (670 µM) has been shown to improve the production of somatic embryos in date palm (*Phoenix dactylifera* L.) and the accumulation of storage proteins [77], which may also be possible if applied to coconut. Although AC is a common additive to coconut SE media, mainly to reduce tissue necrosis [13], it has been demonstrated to reduce the effective concentration of PGRs in the media, due to its adsorptive properties [71]. Therefore, it is crucial to understand the full impact of using AC in a coconut tissue culture media before drawing any conclusions concerning the role a particular PGR may play in the somatic embryogenesis of coconut. In vitro studies on various PGRs for callus induction and embryo maturation of coconut are summarized in Table 1.

## 5. Acclimatization of Plantlets

A well-established acclimatization protocol is required for the successful plantlet establishment in the field but, to date, knowledge gaps still exist, and hence the acclimatization step is considered to be one of the major limitations for coconut clonal plantlet production [78,79]. Due to the limited number of studies on the acclimatization of SE-derived coconut plantlets, the following information is based on in vitro-derived plantlets coming from embryo culture. Any plantlet coming from tissue culture must be acclimatized by the gradual introduction to new environmental factors, such as an increased light intensity, lower atmospheric relative humidity, greater fluctuations in daytime temperatures, and other biotic stresses [80]. The acclimatization process for coconut can take up to 3 months [81]; however, some coconut plantlets display restricted growth after being transferred to nursery conditions [82].

Light intensity is one of the most significant factors that can affect the success of acclimatization of in vitro-derived coconut plantlets [83]. Prior to the commencement of acclimatization, exposure to a higher light intensity has been shown to improve the final survival rate of embryo culture-derived plantlets [84]. An appropriately high photosynthetic activity in natural-grown coconut seedlings was found at a photosynthetic photon flux density (PPFD) of ca.1400 to 1700 μmol m^−2^ s^−1^, but it varied depending on leaf age, plant nutrition, and other climate variables [85]. Assy Bah et al., 1987 [86] used a light intensity of 3000 Lux (ca. PPFD 40 μmol m^−2^ s^−1^) to acclimatize in vitro coconut plantlets from the zygotic embryo culture.

High humidity is required in the initial weeks of acclimatization to continue growth stimulation of in vitro plantlets [87]. However, the atmospheric relative humidity (RH) needs to be progressively reduced to match the humidity the plantlets will experience once planted in the field. To achieve this, plantlets in vitro are usually exposed to the atmosphere of the culture room, then moved in a time-orderly fashion into a humidity chamber (e.g., a plastic tent or wooden box humidity chamber). Such treatments improved survival of both SE and embryo culture-derived plantlets when compared to a misting chamber [65,87]. A mini growth chamber has been used in the ex vitro rooting and acclimatization of Kopyor coconut seedlings. This glass chamber was able to acclimatize 40 seedlings at the same time [88] and increased the survival rate of the in vitro-derived coconut seedlings by more than 80% when compared to the standard nursery approaches that yielded around 63% survival [65]. In summary, the gradual optimization of ex vitro environmental factors from those experienced in vitro will increase the survival rate of the seedlings during the acclimatization process.

The carbon source and concentration applied at different growth stages under in vitro conditions are also crucial for survival of coconut seedlings. Triques et al., 1997 [82] reported that development of both in vitro-produced coconut seedlings and natural coconut seedlings was stimulated by a sucrose-free medium as compared to a sucrose-containing medium. In 2005 and 2014, Samosir and Adkins [78,89] reported that a reduced sugar (43.8 mM or lower) medium with an enriched atmospheric CO_2_ concentration (1600 ppm) was effective in accelerating the development of coconut seedlings.

Plantlets transitioning through the acclimatization process are highly susceptible to dehydration due to cell wall composition and stomata that are continuously open [89]. The survival of seedlings in vitro mainly depending on the development of cuticle and waxes, and maintain the water balance through the regulation of transpiration via stomata [90]. The studies on the effective regulation of stomata suggested that the plants need a proper balance between photosynthesis and water loss [91]. Photosynthetic rate is strongly affected by temperature [92]. Temperature is one of the crucial elements influencing physiology and development of plantlets [92,93]. However, the influence of temperature has been poorly understood in the acclimatization of coconut plantlets though it directly affects the photosynthetic rate and water loss. 

The middle stages of coconut seedling acclimatization require the transition to a medium that contains the appropriate PGRs to encourage root formation, such as 1-naphthaleneacetic acid (NAA). Triques et al., 1997 [94] achieved 92% rooting in embryo cultured coconut seedlings by applying 1 mg L^−1^ NAA. While Nwite et al., 2017 [95] reported that a medium containing 1.5 mg L^−1^ NAA was beneficial for rooting of micropropagated coconut plantlets. Combinations of PGRs have also been found to be effective. For example, Karun et al., 1998 [96] applied 1 mg L^−1^ NAA and 5 mg L^−1^ 3-Indolebutyric acid (IBA), which produced a better root system in the embryo cultured seedlings than either PGR alone.

In addition to the above factors, the growth stages of the in vitro seedlings when commencing the acclimatization process also affects the survival rate. Ex vitro factors, such as plantlet height and root length, can go on to significantly influence the further development and survival of coconut seedlings during field planting [97]. For example, Kopyor plantlets greater than 20 cm tall with an individual root length of 5 cm or more are required for the best survival rate in the field. Similarly, Fernando et al., 2004 [98] reported that coconut SE plantlets from plumule culture needed to have at least two to three photosynthetic leaves and a mature root system if soil establishment was to be successful.

## 6. Molecular Control of Somatic Embryogenesis in Coconut

Coconut is a highly recalcitrant species and is not easily amenable to most tissue culture approaches [99]. An understanding of the molecular mechanisms that underpin SE in this species will provide positive assistance to the positive manipulation of this process. Several molecular events have been observed to occur in coconut cells during SE and identifying the genes involved should lead to a better understanding of the process of SE, and in turn, help to improve the process. Perez-nunez et al., 2009 [100] identified an ortholog of the *SOMATIC EMBRYOGENESIS RECEPTOR-like KINASE* (*SERK*) gene in coconut which is believed to be important in the early stages of SE. The coconut *CnSERK* gene is expressed in the callus tissues during the induction of SE and has been suggested to be a potential marker for SE induction in coconut [100]. Cyclin-Dependent Kinases (CDKs) are involved in the control of the cell cycle, and a coconut *CnCDKA* gene was isolated by analyzing the previously identified sequences from other plant species [36]. The expression of the *CnCDKA* gene increased during the formation of the embryogenic callus and decreased in the germinated somatic embryos [36]. It is thought that study of the role of CDKs in coconut cells will provide more information on the embryogenic competence of any in vitro cultured cells. KNOTTED-like homeobox (KNOX) proteins have been implicated in plant growth, development, cell specification, and pattern formation in several species. The coconut *CnKNOX1* and *CnKNOX2* genes have been identified through sequence analysis [74]. The *CnKNOX1* and *CnKNOX2* genes were shown to be highly expressed at the coleoptilar and globular stages of somatic embryo growth, respectively. Gibberellic acid increased the expression of *CnKNOX1* and decreased the expression of *CnKNOX2* [74]. Treatment of coconut somatic embryogenic calli with A23187 ionophore promoted the rate of SE by increasing the somatic embryo formation and plantlets, and by altering the expression of the *SERK* gene [101]. Modulating the expression of these genes using exogenous chemicals may be very useful to enhance the development of coconut SE.

Recent developments in “next generation” sequencing technologies are playing an important role in the molecular understanding of several processes, including those of SE. Transcriptome analysis has identified several genes that are involved in various stages in the development of coconut somatic embryos [6,102]. The expression of the *CLAVATA* (*CLV*) gene was increased during the early stages of callus formation, and at least eight other genes were highly expressed in embryogenic calli, including *SERK*, *MITOGEN-ACTIVATED PROTEIN KINASE* (*MAPK*), *APETALA2/ETHYLENE RESPONSIVE FACTOR* (*AP2/ERF*), *SAUR* family protein, *EMBRYOGENIC CELL PROTEIN* (*ECP*), *LATE EMBRYOGENESIS-ABUNDANT PROTEIN* (*LEA*), *ARABINOGALACTAN PROTEIN* (*AGP*), and *AINTEGUMENTA* (*ANT*) [102]. During the somatic embryo development stage, the *GERMIN-LIKE PROTEIN* (*GLP*), *GLUTATHIONE S-TRANSFERASE* (*GST*), *PICKLE* (*PKL*), *WUSCHEL* (*WUS*), and *WRKY* genes were also highly expressed [102]. Micro RNAs (miRNAs) are the modulators of gene expression and affect many cellular processes. Through RNA-sequencing, several coconut miRNAs, and their targets have been found to be present in embryogenic and non-embryogenic calli produced from plumular explants. This provides information on the gene regulatory network during coconut SE [6,103]. In all plant species, epigenetic mechanisms are important for cellular programming and have been shown to be important during SE of coconut [104]. Pre-treatment with an inhibitor of DNA methylation (5-azacytidine; AzaC) promoted the early somatic embryo formation from four- to 10-fold and modulated the expression of several genes that are involved in the SE of coconut, viz., *SERK*, *WUS*, *BBM*, and *LEC* [104]. Recently, a draft nuclear genome sequence for both Tall and Dwarf coconut types was published, which will greatly increase the understanding of the genes involved in coconut SE [105,106]. Gene expression studies during the somatic embryogenesis in coconut are summarized in Table 2.

## 7. Future Prospects

Twenty years ago, Punchihewa 1999 [107] stated “the poor genetic makeup of the coconut planting materials used some 70 years ago was one significant reason for the present day low fruit production” and that the availability of high quality planting materials was reducing the rate of palm replanting. Twenty years later, the situation remains unchanged, and very little or no replanting of selected genetic types is occurring worldwide [5]. However, during the same period of time, the market demand for coconut and its products has increased more than five-fold. This clearly indicates the need for an alternative method for the rapid production of planting materials from genetically superior stock, to meet the ever-growing demand for coconut products. In addition to the ageing of palms, production is also affected by several biotic and abiotic stresses, and the crop is susceptible to certain natural disasters. The traditional methods of seedling production from fruit are not sufficient to meet the current needs for planting material. Therefore, clonal propagation via SE is a preferred alternative method. It is hoped that such technology will rapidly produce true-to-type, early bearing, pest- and disease-resistant plantlets, in large quantities. The success of SE will be dependent on the palm genotype, explant type, PGRs, medium, and the acclimatization procedure. A firm molecular understanding of the process of SE is certainly a key for further improvements in this technology. Due to the recent developments in the genomic sequencing, several genes, miRNAs, and their target genes have been identified during the process of SE in coconut, and further studies are now needed to ensure this new information is used to manipulate the current protocols.

Recently, it has been reported that the current SE protocol did not change the genetic make-up of a cloned coconut plantlet [54] as no variation was detected in the plantlets produced under in vitro conditions. The genetic fidelity experiment of the plantlets indicated that the SE protocols employed, although not yet efficient, should be suitable for the large-scale production of cloned plantlets. At this point in time, one biofactory facility for coconut plantlet production has been established in Mexico using the available SE protocol at a semi-commercial scale [108]. Field trials have been conducted with the SE-derived plantlets, and performance determined to be good, including growth and development to the fruit-bearing stage. These SE-cloned plants started bearing fruits about 6 months earlier than plants produced from seed.

Public–private partnerships are important to take this technology to a commercial scale. Production of quality plantlets via SE, for the replacing of the 5 billion senile coconut palms around the world, has the potential to meet this demand, and will also increase the income of smallholder coconut farmers around the world. 

## Figures and Tables

**Table 1:** In vitro studies on explant, media composition, and plant growth regulators in order from older to recent studies in coconut.

Explant	Media Composition and Plant Growth Regulators			Variety/Hybrid	References
	Basal Media (Callus Induction)	Plant Growth Regulator (Callus Induction)	Plant Growth Regulator (Embryo Maturation)		
Rachilla and stem	Eeuwen’s Y3 basal medium and sucrose (6.8%), and agar (0.39%)	2,4-D (0.1 μM), BAP (5 μM), and GA_3_ (10 μM)	BAP	Jamaican Malayan Dwarf	[18]
Young foliage tissue	Eeuwens’ salts, Morel’s Vitamin, sucrose (30 g/L) and agar (0.8%)	2,4-D or TCPP	BAP	Malayan Yellow Dwarf (MYD) × West African Tall (WAT)	[45]
Root, stem and leave	Murashige and Skoog (MS) macro-nutrients, Y3 micro-nutrients, improved Blake vitamin, sucrose (5%), Activated Charcoal (AC; 0.25%), 300 mgL^−1^ casein hydrolysate and myo-inositol (100 mgL^−1^), 5% agar	2,4-D (100 μM), BAP (5 μM) and 2iP (5 μM)	2,4-D (100 μM), BAP (5 μM) and 2iP (5 μM)	Jamaican Malayan Dwarf	[55]
Rachilla, stem and foliage	Eeuwen’s Y3, sucrose (5%), and AC (0.25%), and agar (0.6%)	2,4-D (452 μM), NAA (2.69 μM), BAP (8.88 μM), Kinetin (4.65 μM)	2,4-D (2.3 μM)	West Coast Tall	[56]
Young embryo	Gamborg’s B5 medium, and agar (0.7%)	IAA, NAA, 2,4-D, BAP or Kinetin (0.5 mg/L to 5 mg/L)	IAA (2 mg/L)	West Coast Tall	[57]
Embryo	Broad spectrum tissue culture medium, sucrose (30 g/L), AC (0.25%) and agar (0.8%)	2,4-D (12–20 μM)	2,4-D (8 μM and 2 μM), BAP (10 μM) and Kinetin (10 μM)	Typica	[33]
Immature inflorescence	Eeuwen’s Y3, Morel and Wetmore (MW) Vitamins, sucrose (116.8 mM) and AC (2 g/L)		2,4-D and BAP (10^−5^ M)	MYD × WAT, WAT × MYD and MYD	[58]
Immature inflorescence	Modified MS macro nutrients, Nitsch micronutrients, MW Vitamins, EDTA (26 mg), iron (24.9 mg), sucrose (20 g/L), ascorbic acid (100 mg/L), malic acid (100 mg/L), adenine sulfate (30 mg/L), agar (7.5 g/L) and AC (3 g/L)	2,4-D (100 mg/L) and BAP (1 mg/ L)	2,4-D (130 mg/L) and BAP (140 mg/L)	PB 121 (MYDxWAT)	[59]
Embryo slice	Eeuwen’s Y3, sucrose (90 mM), AC (2.5 g/L) and Agar (0.7%)	2,4-D (125 μM), AVG (1 µM) and	2,4-D (50 μM)	Batu Layar Tall	[60]
Plumule	Eeuwen’s Y3, AC (2.5 g/L) and gelrite (3 g/L)	2,4-D (0.1 mM)	2,4-D (1 μM) and BAP (50 μM)	Malayan Dwarf	[41]
Mature embryo slice	M2, sucrose (0–100 g/L), and AC (2.5 g/L), and agar (7.5 g/L)	2,4-D (125 μM) and ABA (0–90 μM)	2,4-D	Batu Layar Tall	[34]
Immature embryo	BM72, sucrose (40 g/L) and AC (0.25%), and agar (0.8%)	2,4-D (24 μM), and ABA (2.5–7.5 μM)	2,4-D and cytokinin (2–10 μM)	Sri Lanka Tall	[20]
Plumule	BM72, sucrose (4% w/v), and agar (0.8%)	2,4-D (24 μM)	N/A	Sri Lanka Tall	[61]
Plumule	Eeuwen’s Y3, sucrose (30 g/L), AC (2.5 g/L), gelrite (3 g/L)	2,4-D (600 μM)	2,4-D (6 μM), and BAP (300 μM)	Green Malayan Dwarf	[11]
Plumule	Eeuwen’s Y3, gelrite (3 g/L) and AC (2.5 g/L)	2,4-D (0.65 mM)	2,4-D (6 μM), and BAP (300 μM)	Green Malayan Dwarf	[62]
Unfertilized ovary	CRI 72 and agar (2%)	2,4-D (100 μM)	2,4-D (66 μM) and ABA (5 μM)	Sri Lanka Tall	[29]
Immature inflorescence	Eeuwen’s Y3, sucrose (30 g/L) and AC (2.5 g/L)	Spermine (0.01 µM), Smoke-saturated-water (10%) and auxin (500 µ M).	2,4-D and PGR free no PGR and	Malayan Yellow Dwarf	[63]
Inflorescence	CRI 72, sucrose (40 g/L) and AC (0.1%)	2,4-D (100 μM) and TDZ (9 μM)	No growth regulators	Sri Lanka Tall	[52]
Rachilla	Eeuwen’s Y3, AC (2.5 g/L) and gelrite (3 g/L))	2,4-D (0.65 mM)	2,4-D (0.325 and 0.006 mM), BAP (0.3 mM) and GA3 (0.0046 mM)	MYD × MXPT Malayan Red Dwarf) MRD × (Tagnanan) TAGT	[40]
Plumule	Eeuwen’s Y3, MW vitamins, agar (2.5 g/L)	2,4-D (600 μM)	2,4-D (6 μM) and BAP (300 μM)	MYD, Makapuno, XXD and PB121	[10]
Plumule	Eeuwen’s Y3, sucrose (50 g/L), AC (2.5 g/L) and gelrite (3 g/L)	2,4-D (600 μM)	2,4-D (6 μM) and (300 μM BAP)	Green Malayan Dwarf	[38]

2,4-dichlorophenoxyacetic acid (2,4-D), 6-benzylaminopurine (BAP), thidiazuron (TDZ), abscisic acid (ABA), gibberellic acid (GA3), indole acetic acid (IAA), naphthaleneacetic acid (NAA), aminoethoxyvinylglycine (AVG).

**Table 2:** Gene expression studies on the somatic embryogenesis of coconut.

Gene Family and Other Genetic Mechanism	Gene(s)	Gene Expression Pattern	References
*SOMATIC EMBRYOGENESIS RECEPTOR-like KINASE* (*SERK*)	*CnSERK*	Highly expressed in the callus tissues at the initiation stage of SE	[100]
*Cyclin-Dependent Kinases* (*CDKs*)	*CnCDKA*	Increased expression during the formation of embryogenic callus and decreased in the germinated somatic embryos	[36]
*KNOTTED-like homeobox* (*KNOX*)	*CnKNOX1*	Highly expressed at the coleoptilar somatic embryo growth	[74]
	*CnKNOX2*	Highly expressed at the globular stages of somatic embryo growth	
*CLAVATA* (*CLV*)	*CLV*	Increased at the early stages of callus formation	[102]
*SERK*, *MITOGEN-ACTIVATED PROTEIN KINASE* (*MAPK*), *APETALA2/ETHYLENE RESPONSIVE FACTOR* (*AP2/ERF*), *SAUR* family protein, *EMBRYOGENIC CELL PROTEIN* (*ECP*), *LATE EMBRYOGENESIS-ABUNDANT PROTEIN* (*LEA*), *ARABINOGALACTAN PROTEIN* (*AGP*) and *AINTEGUMENTA* (*ANT*)	*SERK*, *MAPK*, *AP2/ERF*, *SAUR*, *ECP*, *LEA*, *AGP*, *ANT*	Highly expressed in embryogenic calli	[102]
*GERMIN-LIKE PROTEIN* (*GLP*), *GLUTATHIONE S-TRANSFERASE* (*GST*), *PICKLE* (*PKL*), *WUSCHEL* (*WUS*)	*GLP*, *GST*, *PKL*, *WUS*, *WRKY*	Highly expressed during the somatic embryo development stage	[102]
Micro RNAs (miRNAs)	Several miRNAs, and their targets	Found in embryogenic and non-embryogenic calli produced from plumular explants	[6,103]
Epigenetic mechanism	*SERK*, *WUS*, *BBM* and *LEC*	Treatment with DNA methylation inhibitor promoted early SE formation and modulated the expression of *SERK*, *WUS*, *BBM* and *LEC* genes	[104]

## Data Availability

Not applicable.
